# Supplementary material for: Processing of different spatial scales in the human brain
Source: eLife. 2019 Sep 10;8:e47492. doi: 10.7554/eLife.47492 (PMC6739872; doi:10.7554/eLife.47492)
Supplement: Supplementary file 1. — Table S1: coordinates of all scene-sensitive activations, sorted by the spatial scale at the position of the Gaussian fit peak. Table S2: relation of cortical gradients to large-scale resting-state brain systems. Table S3: verbal descriptions of strategy used in task performance for each spatial scale. [file elife-47492-supp1.docx]

| MNI coordinates | | | Activation size (voxels) | Region name (AICHA atlas) |  | MNI coordinates | | | Activation size (voxels) | Region name (AICHA atlas) |
| --- | --- | --- | --- | --- | --- | --- | --- | --- | --- | --- |
| X | **y** | **z** |  |  |  | **x** | **y** | **z** |  |  |
| Scale 1 – room | | | | |  | Scale 5 - country | | | | |
| 58 | -41 | 30 | 88 | Supramarginal gyrus R |  | 46 | -67 | 36 | 4 | Angular gyrus R |
| -59 | -32 | 39 | 115 | Supramarginal gyrus L |  | -46 | -67 | 33 | 11 | Angular gyrus L |
| -14 | -65 | 53 | 370 | Superior parietal gyrus L |  | -1 | -52 | 20 | 70 | Precuneus L |
| 12 | -62 | 57 | 120 | Precuneus R |  | 25 | -82 | -9 | 8 | Fusiform gyrus R |
| 13 | -35 | 46 | 24 | Cingulate sulcus R |  | 16 | -10 | -17 | 11 | Parahippocampal gyrus R |
| -12 | -32 | 44 | 33 | Cingulate sulcus L |  | -18 | -13 | -15 | 12 | Parahippocampal gyrus L |
| 47 | -55 | 16 | 5 | Superior temporal sulcus R |  | 45 | 32 | 20 | 6 | Inferior frontal sulcus R |
| 24 | 2 | 59 | 59 | Superior frontal sulcus R |  | 19 | 33 | 45 | 28 | Superior frontal sulcus R |
| 58 | -55 | 2 | 89 | Middle temporal gyrus R |  | 19 | 68 | 12 | 20 | Superior frontal sulcus R |
| -52 | -60 | 7 | 274 | Middle temporal gyrus L |  | 12 | 69 | -2 | 5 | Superior frontal sulcus R |
| 31 | -47 | -8 | 45 | Fusiform gyrus R |  | -13 | 64 | 16 | 5 | Superior frontal gyrus L |
| -30 | -51 | -9 | 50 | Fusiform gyrus L |  | -19 | 34 | 45 | 4 | Superior frontal sulcus L |
| -25 | 3 | 56 | 152 | Superior frontal sulcus L |  | -20 | 60 | 18 | 6 | Superior frontal gyrus L |
| 31 | 42 | 29 | 21 | Middle frontal gyrus R |  | -2 | 56 | -5 | 114 | Anterior rostral sulcus L |
| -37 | 34 | 28 | 6 | Middle frontal gyrus L |  | 30 | 34 | -10 | 12 | Orbital sulcus R |
| 43 | -75 | 22 | 63 | Middle occipital gyrus R |  | -32 | 27 | -18 | 9 | Inferior orbitofrontal gyrus L |
| -33 | -79 | 27 | 171 | Middle occipital gyrus L |  | -40 | 43 | -11 | 7 | Middle orbitofrontal gyrus L |
| Scale 2 – building | | | | |  | 30 | -88 | 8 | 71 | Lateral occipital gyrus R |
| -45 | -71 | 24 | 92 | Angular gyrus L |  | -22 | -92 | -1 | 15 | Lateral occipital gyrus L |
| -18 | -67 | 20 | 36 | Parietooccipital sulcus L |  | -25 | -92 | 11 | 20 | Lateral occipital gyrus L |
| -24 | -56 | 9 | 3 | Parietooccipital sulcus L |  | -33 | -84 | -2 | 5 | Lateral occipital gyrus L |
| 21 | -61 | 22 | 36 | Precuneus R |  | 17 | -94 | -5 | 16 | Calcarine gyrus R |
| -1 | -54 | 51 | 142 | Precuneus L |  | -3 | 16 | -7 | 3 | Caudate nucleus L |
| -7 | -75 | 46 | 4 | Precuneus L |  | -41 | -72 | -39 | 19 | Cerebellum |
| -62 | -57 | 18 | 7 | Superior temporal sulcus L |  | Scale 6 - continent | | | | |
| 28 | -43 | -8 | 34 | Parahippocampal gyrus R |  | 40 | -59 | 43 | 104 | Inferior parietal gyrus R |
| -29 | -43 | -10 | 63 | Parahippocampal gyrus L |  | -45 | -70 | 38 | 5 | Angular gyrus L |
| 42 | -76 | 30 | 43 | Middle occipital gyrus R |  | 0 | -40 | 31 | 215 | Posterior cingulate gyrus |
| 17 | -46 | -48 | 8 | Cerebellum |  | -56 | 7 | -9 | 3 | Superior temporal gyrus L |
| Scale 3 – neighborhood | | | | |  | 62 | -40 | -8 | 6 | Middle temporal gyrus R |
| 18 | -47 | 3 | 261 | Parietooccipital sulcus R |  | -29 | -87 | -9 | 362 | Fusiform gyrus L |
| -14 | -54 | 7 | 274 | Precuneus L |  | 27 | 21 | 47 | 16 | Superior frontal sulcus R |
| scale 4 – city | | | | |  | 23 | 64 | 6 | 18 | Superior frontal sulcus R |
| 11 | -50 | 10 | 66 | Precuneus R |  | -13 | 43 | 41 | 20 | Superior frontal gyrus L |
| -9 | -52 | 11 | 44 | Precuneus L |  | 46 | 27 | 24 | 30 | Inferior frontal sulcus R |
| 23 | -19 | -19 | 62 | Parahippocampal gyrus R |  | -2 | 66 | 6 | 8 | Anterior rostral sulcus L |
| 22 | -35 | -19 | 11 | Parahippocampal gyrus R |  | 35 | 37 | -13 | 29 | Orbital sulcus R |
| -20 | -35 | -16 | 8 | Parahippocampal gyrus L |  | -33 | 35 | -14 | 39 | Orbital sulcus L |
| -23 | -19 | -18 | 45 | Hippocampus L |  | -6 | 45 | -10 | 30 | Medial orbitofrontal gyrus L |
| 23 | 27 | 39 | 6 | Superior frontal sulcus R |  | -3 | 58 | -11 | 4 | Medial orbitofrontal gyrus L |
| 6 | 62 | -5 | 21 | Medial orbitofrontal gyrus R |  | 32 | -86 | -5 | 346 | Lateral occipital gyrus R |
| -20 | -97 | 13 | 12 | Lateral occipital gyrus L |  | 42 | -70 | -38 | 14 | Cerebellum |
|  |  |  |  |  |  | -13 | -83 | -34 | 4 | Cerebellum |

**Table S1: coordinates of all scene-sensitive activations, sorted by the spatial scale at the position of the Gaussian fit peak.** Activation clusters with less than 3 voxels are not shown. Labels are by the AICHA parcellation atlas. Coordinates indicate location of center of mass for each cluster.

|  | **Medial parietal gradient** | | | | |  | **Medial temporal gradient** | | | | | |  | **Lateral occipito-parietal gradient** | | | |
| --- | --- | --- | --- | --- | --- | --- | --- | --- | --- | --- | --- | --- | --- | --- | --- | --- | --- |
|  | Building | Neighborhood | City | Country | Continent |  | Room | Building | Neighborhood | City | Country | Continent |  | Room | Building | Country | Continent |
| **Visual network** | **77%** | 46% | 23% | 0% | 0% |  | **99%** | **96%** | **88%** | 30% | N/A | N/A |  | **45%** | 14% | 0% | 0% |
| **Default-mode network** | 19% | **54%** | **77%** | **100%** | **88%** |  | 0% | 3% | 12% | **60%** | N/A | N/A |  | 7% | **40%** | **98%** | **43%** |
|  |  |  |  |  |  |  |  |  |  |  |  |  |  |  |  |  |  |
| **Dorsal attention network** | 0% | 0% | 0% | 0% | 0% |  | 1% | 1% | 0% | 0% | N/A | N/A |  | 47% | 40% | 0% | 27% |
| **Fronto-parietal control network** | 4% | 0% | 0% | 0% | 10% |  | 0% | 0% | 0% | 0% | N/A | N/A |  | 2% | 4% | 2% | 29% |
| **Medial temporal-orbitofrontal network** | 0% | 0% | 0% | 0% | 0% |  | 0% | 0% | 0% | 10% | N/A | N/A |  | 0% | 0% | 0% | 0% |
| **Ventral attention network** | 0% | 0% | 0% | 0% | 2% |  | 0% | 0% | 0% | 0% | N/A | N/A |  | 0% | 1% | 0% | 0% |
| **Sensorimotor network** | 0% | 0% | 0% | 0% | 0% |  | 0% | 0% | 0% | 0% | N/A | N/A |  | 0% | 0% | 0% | 0% |

**Table S2: relation of cortical gradients to large-scale resting-state brain systems.** The percent of overlap between voxels of each scene-selective region of the three cortical gradients and seven large-scale resting-state brain networks, as previously identified (Yeo et al., 2011). Within each of the three gradients, a shift can be seen from the visual network that overlaps with the smaller spatial scales to the default-mode network that overlaps with the larger spatial scales. Relation to resting-state networks for the two largest scales in the medial temporal gradient could not be determined, as they encompass regions not included in the brain mask used in Yeo et al., 2011.

|  | **Room** | **Building** | **Neighborhood** | **City** | **Country** | **Continent** |
| --- | --- | --- | --- | --- | --- | --- |
| Looking from a specific fixed point in the environment and comparing distances to two locations | 12 / 18 | 3 / 18 | 3 / 18 | 3 / 18 | 1 / 18 | 1 / 18 |
| Imagining a map-like view of the location | - | 1 / 18 | 5 / 18 | 9 / 18 | 11 / 18 | 13 / 18 |
| Comparing walking / driving / flying time to the two locations | 1 / 18 | 10 / 18 | 8 / 18 | 4 / 18 | 3 / 18 | 2 / 18 |
| Drawing imaginary lines between the locations and comparing their length | 2 / 18 | - | 2 / 18 | 2 / 18 | 2 / 18 | 1 / 18 |
| Answering based on knowledge / feeling of what is closer | 2 / 18 | 3 / 18 | 4 / 18 | 4 / 18 | 4 / 18 | 2 / 18 |

**Table S3: verbal descriptions of strategy used in task performance for each spatial scale**. The number of subjects that reported using each strategy at each scale, out of 18 subjects who filled the questionnaire (one subject did not provide strategy descriptions).
